# Estrogen response in luminal breast cancer

**DOI:** 10.18632/oncotarget.1354

**Published:** 2013-09-21

**Authors:** Leticia B. A. Rangel, Tim Hui-Ming Huang

**Affiliations:** University of Texas Health Science Center at San Antonio, TX, USA and Universidade Federal do Espirito Santo, ES, Brazil/CNPq (Ciencias sem Fronteiras)

Estrogen receptor α (ERα) is the defining and driving transcription factor in luminal breast cancers and genes modulated by ERα can dictate cell growth and endocrine response. Nonetheless, genomic mechanisms which govern ERα-driven tumorigenesis, acquisition of chemoresistant phenotype and tumor metastases remain elusive. Recently, scientists have explored to shed light on ER regulated genomic events in primary breast cancer with divergent clinical outcome and in distant ERα+ metastases. Mapping of genome-wide ER binding events by chromatin immunoprecipitation followed by high-throughput sequencing revealed differential FoxA1-mediated ERα-chromatin binding programming that results in predictive gene signatures exclusive for ERα+ breast cancer clinical outcome, and is characterized by remarkable intensification of ERα binding signal in tumors that progress towards a poor prognosis [1,2]. Furthermore, ERα-chromatin interactions occur regardless of tumor endocrine therapy sensitivity. Nevertheless, there is differentially stronger ERα binding signal in tamoxifen resistant in comparison with tamoxifen sensitive lineages [1]. Although the mechanisms underlying ERα binding plasticity in breast cancer remain to be elucidated, the influence of specific stimuli, as those triggered by growth factors pathways [3,4], may result in differential ERα binding patterns that regulate gene expression programs, sensitivity to endocrine therapy and overall clinical outcome in ERα+ breast tumors. It is worthwhile to emphasize that the only DNA motif found enriched in the core of ER binding events is the estrogen response element (ERE) [1].

Of note, innovative model in ERα-driven tumorigenesis and cancer aggressiveness has emerged from the concept of the tag-team model of gene expression in luminal breast cancer, according to which amplified distant EREs (DEREs) coordinately and remotely modulates the transcription of distant genes through long-rage chromatin interactions [5]. The model hypothesis highlights DERE axes as hot spots for concomitant and aberrant genome amplification in luminal breast cancer; thus coordinately and persistently deregulating target transcriptome, including co-amplification of oncogenes and repression of tumor-suppressor loci for cancer development and endocrine therapy resistance. Using integrated next-generation sequencing approaches, two densely ERα-bound DERE regions, frequently amplified in ERα+ luminal breast cancers, were mapped on chromosomes 17q23 and 20q13 [5]; genomic amplification of which has been associated with endocrine therapy relapse and overall poor prognosis in breast cancer [6]. Moreover, integration of 3C dataset with published time-course study of gene expression revealed 95 loci remotely interacting with 20q13 DEREs, 38 genes with 17q23 DEREs, and 46 estrogen-responsive targets [5]. Interestingly, a strong body of evidences has demonstrated the novel role of the DEREs targeted genes *THRAP1* and *ZIM2* as tumor suppressors in luminal breast cancer. It is reasonable to speculate that the increased frequency of chromatin interactions might play a role to elicit epigenetic repression of DERE-regulated genes. The aforementioned phenomenon derives from genomic alterations induced by chronic estrogenic exposure of mammary cells that persist in breast progenitor cells [5], and progressively accumulate in malignant differentiating cells, in agreement with previous findings that points to the intensification of the ERα binding signal and ERE amplification during breast cancer worsening prognosis [1,2].

Precisely, these findings open new avenues to explore amplified DEREs in 17q23 and 20q13 as potential prognostic markers in luminal breast cancer, based on the rationale that the aberrant amplification of DEREs may enable functionality of residual estrogen/ERα regardless of the administration of selective ER blockers, such as tamoxifen [5]. This concept, at least partially, can explain the benefit of using aromatase inhibitors in post-menopausal women, which present the higher incidence of luminal breast cancer. The inhibition of the main source of estrogen production in these patients may prevent the E2/ERα-induced anomalous amplification of DEREs. On the other hand, the amplified DEREs axes and the tag-team model of gene expression modulation in luminal breast cancer currently challenges the investigators to determine the precise factors and mechanisms that trigger the malignant transformation of normal progenitor mammary cells. Amplified DNA regulatory elements may result from sustained amplification of DERE-DERE interactions that may intensify chromatin interaction within regions harboring clustered breakpoints, exquisitely prone to genomic rearrangements, as a result of genomic instability [5,7]. During neoplastic transformation, defects in double-strand break repair may destabilize these physical interactions, promoting insertions and self-duplication of 20q13 DERE clusters, for example, into regions of seven derivative chromosomes [5]. In contrast to this concept, it is puzzling that mammary cells sustain the benign phenotype in the majority of women regardless of the estrogenic milieu. At present, it is also important to evaluate the occurrence of aberrant DERE amplification and its peculiarities in controlling target genes expression in ovarian cancer, commonly lethal and tamoxifen resistant. Nonetheless, cumulative evidence hint the potential roles of the amplified distant DNA response elements axes and the tag-team model of gene expression modulation in hormone-driven carcinogenesis and cancer progression. More importantly, knowledge gained out of the study [5] should be instrumental for further understanding of cancer genomics and bringing of innovative interventions to control cancer.
